# Unlocking the clinical potential of paired inspiratory and expiratory CT scans in the differential diagnosis of cystic lung diseases: A systematic review

**DOI:** 10.1371/journal.pone.0314572

**Published:** 2024-12-03

**Authors:** Lucas Gabriel R. Pinheiro, Carlos Augusto Treviso, Gabriele Carra Forte, Enrico Mattana Muller, Bruno Hochhegger, Rubens Gabriel Feijó Andrade

**Affiliations:** 1 Pontifícia Universidade Católica do Rio Grande do Sul, Porto Alegre, Rio Grande do Sul, Brazil; 2 University of Florida, Gainesville, FL, United States of America; University of Turin: Universita degli Studi di Torino, ITALY

## Abstract

**Introduction:**

Currently, high-resolution computed tomography (HRCT) is the imaging of choice for the differential diagnosis of various cystic lung lesions, including true cystic lung diseases (CLD) and lesions that may mimic them. However, the traditionally used inspiratory scan still presents a significant spectrum of overlapping radiological features. Recent studies have demonstrated variation in lesion size between inspiratory and expiratory phases, probably due to cyst-airway communication. In this study, we aimed to conduct a systematic review of paired inspiratory and expiratory HRCT in the assessment of cystic lesions as an additional tool to narrow the differential diagnosis.

**Methods:**

A systematic search was performed in PubMed, Scopus, EMBASE, BVS, and Cochrane through August 2023. Full-text articles that performed paired inspiratory and expiratory CT scans in adult patients with cystic lung lesions were included, with the outcome measured as the reduction in lesion size according to the respiratory phase. Diagnoses were confirmed through histopathological or radiological features.

**Results:**

Out of the 96 records, three studies met the criteria for inclusion and were analyzed, comprising a total of 149 participants and 513 cystic lesions. Pulmonary Langerhans Cell Histiocytosis (PLCH), Lymphangioleiomyomatosis (LAM) honeycombing and cystic bronchiectasis became considerably smaller during expiratory CT scans, while the size of emphysema tended to remain constant during respiratory cycles.

**Conclusions:**

This study has suggested that paired inspiratory and expiratory CT scans can be valuable for helping differentiate between emphysema and other diseases with a cystic pattern due to their ability to reveal dynamic properties of the lesions. However, the average reduction in cyst size as a single parameter is not sufficient for further refining diagnostics. Studies exploring advanced metrics to assess the reduction in lesion diameter emerge as potential opportunities to investigate the cyst-airway communication hypothesis and further enhance the diagnostic accuracy of paired methods.

## Introduction

Cystic lung diseases (CLDs) refer to a collection of heterogeneous conditions characterized by the existence of numerous, thinly-walled, air-filled spaces within the pulmonary parenchyma, often involving more than one pulmonary lobe [1]. A differential diagnosis of CLDs includes a wide range of diseases, including neoplastic, congenital, genetic, developmental, lymphoproliferative, infectious and inflammatory [2]. Lymphangioleiomyomatosis (LAM), pulmonary Langerhans cell histiocytosis (PLCH), Birt-Hogg-Dubé syndrome (BHD), and lymphoid interstitial pneumonia (LIP) are considered the main true cystic diseases [3, 4]. However, the diagnostic scope may also include other rare conditions, such as amyloidosis, light chain disease, hypersensitivity pneumonitis, Pneumocystis pneumonia, cystic metastasis, and papillomatosis [3, 4]. Emphysema, bullae, bronchiectasis, pneumatoceles, and honeycombing also produce low attenuation lesions, however, they do not meet the criteria for true CLD [2, 4].

For clinical practice, it is crucial to differentiate true CLDs from common abnormalities like emphysema and benign age-related pulmonary cysts, which requires careful analysis of imaging findings and patient history [5]. Age-related cysts are typically solitary or found in small numbers in asymptomatic individuals over 40 years old, often located peripherally in the lower lobes with well-defined margins. Emphysema may show multiple cyst-like areas but is characterized by air trapping and destruction of alveolar walls, linked to smoking history [4, 5]. True CLDs, on the other hand, present demographic characteristics and extrapulmonary findings that help establish the diagnosis. For instance, PLCH typically affects young adult smokers, presenting with irregular, thick-walled cysts predominantly in the upper lobes [3, 4]. LIP occurs in middle-aged adults and is characterized by ground-glass opacities and cysts distributed around bronchovascular bundles and in the lower lobes. LAM primarily affects women of childbearing age, showing diffuse and symmetrical cysts of similar size throughout the lungs. BHDS features cysts of variable sizes, often subpleural and peri-fissural, with a lower lobe predominance [3].

Currently, high-resolution computed tomography (HRCT) is the imaging of choice for differential diagnosis of lung diseases with a cystic pattern, including true CLDs and all lesions that may mimic them [6]. The method has a high accuracy in identifying the distribution, size, extent, and regularity of cysts, as well as extrapulmonary changes [6]. However, although several cystic lung diseases often exhibit a characteristic appearance that allows their confirmation on HRCT, there exists a spectrum of imaging findings with variable degrees of overlap, making radiological diagnosis challenging and maintaining the need for pulmonary biopsy to establish a definitive diagnosis in cases where there is uncertainty and confirmation is crucial for management [7].

The characterization of cysts by HRCT traditionally occurs during the inspiratory phase. However, while this approach significantly narrows the differential diagnosis, it provides only a static view of lung morphology [8]. Expiratory CT imaging is an additional acquisition performed with the patient in a supine position and acquired at end-expiration, allowing for the identification of air trapping. Combining both inspiratory and expiratory CT provides a way to assess dynamic variations in lung attenuation resulting from interactions among alveolar air volume, pulmonary interstitium, and blood volume [8]. In current clinical practice, expiratory CT is already employed in the evaluation of Chronic Obstructive Pulmonary Disease (COPD), especially for patients with small airway diseases and resulting pulmonary emphysema who experience lung hyperinflation and air trapping [9–12].

Recent studies have indicated that cyst size may vary to different levels when HRCT is conducted during both the expiratory and inspiratory phases, possibly due to differing degrees of communication between the cystic lesion and the airway [13–15]. In this study, we aimed to conduct a systematic review of paired inspiratory and expiratory CT in the assessment of cystic lung diseases as an additional tool to narrow the differential diagnosis.

## Methods

The Preferred Reporting Items for Systematic Reviews (PRISMA) and Enhancing the Quality and Transparency of Health Research (EQUATOR) Reporting Guidelines were used to conduct this study.

### Search strategy and study selection

A systematic search was performed in different databases including PubMed (U.S. National Library of Medicine), Scopus (Elsevier), Embase (Elsevier), BVS, and Cochrane through August 2023. Studies were selected based on the PICO (population, intervention, comparator, and outcome) approach. The target population consisted of adults (≥18 years) previously diagnosed with cystic lung disease confirmed through histopathological or radiological features. Conditions that cause low-attenuation lesions but are not primarily classified as cystic, particularly emphysema and honeycombing, were included in several studies alongside more typical cystic lesions. Given their relevance to the differential diagnosis, these diseases were included within the scope of CLDs and, consequently, did not serve as an exclusion criterion for this review.

The intervention was paired inspiratory and expiratory CT scans. The existence of a comparison group with isolated expiratory CT or isolated inspiratory CT was not necessary. The outcome measured was the cyst size in each of the CT phases. The study design included cross-sectional and cohort studies. The "Snowball Method" was utilized to identify additional publications from the reference lists of relevant articles. Case reports, books, editorials, abstracts, and duplicates, as well as studies that did not follow the PIRO criteria, were excluded. The search was conducted without language restriction. The search terms were *Paired inspiratory-expiratory*, *expiratory CT*, *expiratory computed tomography*, *expiratory multisection CT*, *expiratory chest CT*, *expiratory*, *Cystic lung disease*, *Cystic Lesions* and *Cystic lung lesions*. The detailed search strategy is presented in S1 File.

Two researchers reviewed the titles and abstracts of retrieved articles and applied inclusion and exclusion criteria. The full texts of qualifying articles were retrieved and reviewed to confirm study eligibility.

### Data extraction

Two analysts applied the PRISMA standards to assess the literature that was approved for study. Information collected from studies included the following: first author, year of publication, study design, nation of patient recruitment, number of patients enrolled, patient characteristics (sex, age), lesion number, technical specifications (including patient position and maneuver, anatomical level of image acquisition in inspiration and expiration, window width and window level and adequate effort), method of confirming CLD, mean diameter decrease of the cyst, inner thoracic diameter variation, and specific information about cyst dimensions for each diagnose (size of cysts in inspiration and expiration). Data extraction was performed independently, and divergent results were resolved by consensus after a review of the source.

### Study quality assessment

Two investigators independently evaluated the included studies’ quality using the updated Quality Assessment of Diagnostic Accuracy Studies (QUADAS-2), and any discrepancies were settled through discussion with a third investigator. Patient selection, index testing, reference standard, and flow and timing are the four components of this quality control system. The third criterion is based on bias issues related to applicability and the danger of bias. Bias risk was rated as being high, low, or unclear.

## Results

**Fig 1:**
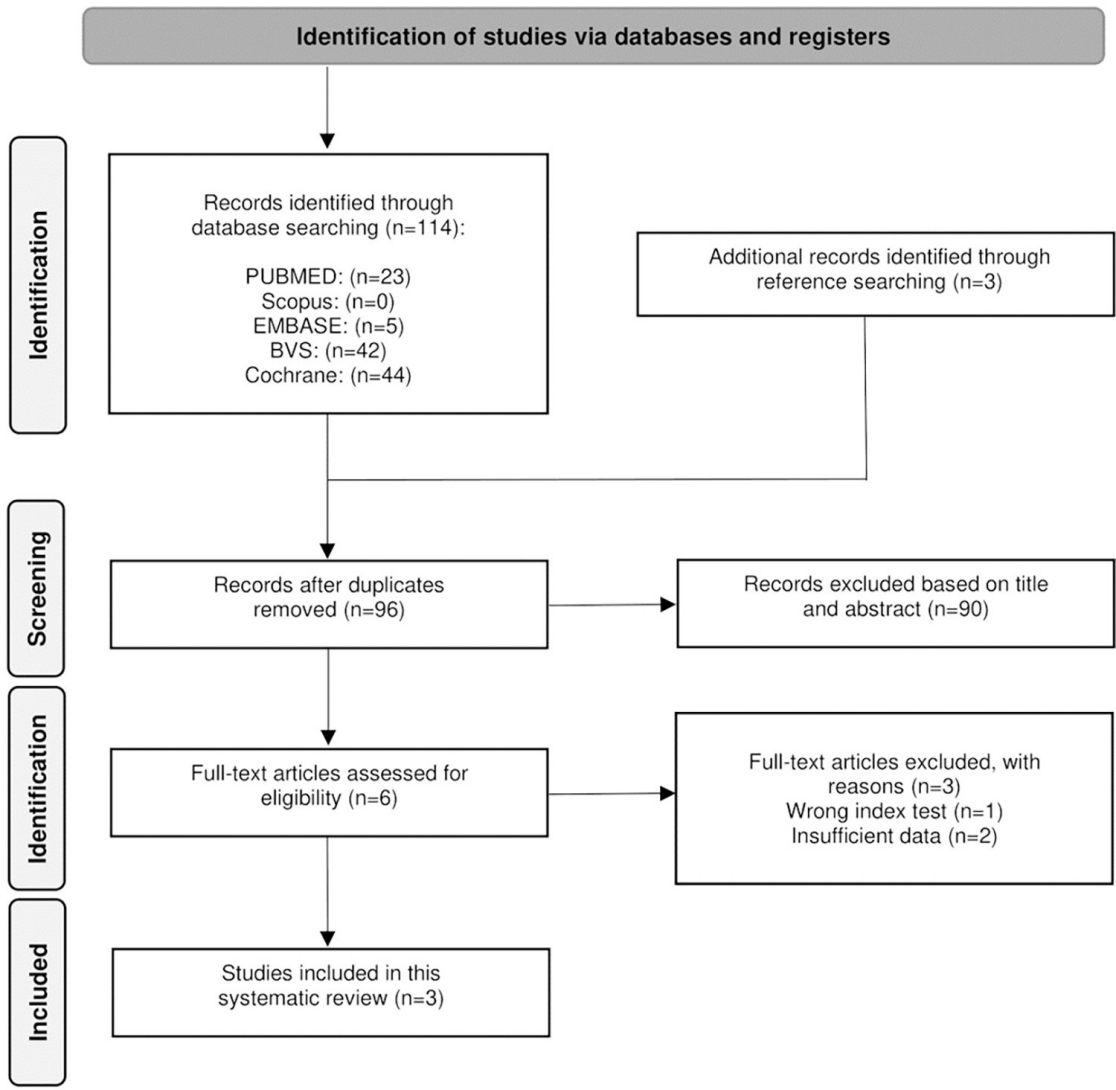
Preferred Reporting Items for Systematic reviews and Meta-Analyses (PRISMA) flow diagram.

In the initial systematic search, 114 studies were identified, and three more records were discovered in additional sources. Following the removal of duplicates, 96 articles remained for title and abstract analysis, and subsequently, six papers were selected for full-text analysis. Based on the eligibility criteria, three items were rejected. Therefore, three studies were analyzed in this systematic review, comprising a total of 149 participants and 513 cystic lesions (Fig 1)

The included studies were published in 1998 [15], 2000 [13], and 2022 [14], respectively, in Canada, South Korea, and the USA. All three studies were retrospective and evaluated inspiratory and expiratory CT scans to compare the size of lesions in different respiratory phases in patients with CLDs. Overall, the population consisted mainly of middle-aged women, and smoking history was included in only one of the studies [14]. Detailed information on patient characteristics is provided in Table 1.

**Table 1:** Main characteristics of the included studies evaluation of cystic lung disease by paired inspiratory and expiratory CT scans.

Study author	Year	Region	n	Men	Age	Study design	Intervention	Mean smoking history*
Hochhegger, B et al	2022	USA	72	43 (59.7%)	46 (49–76)	Retrospective	Paired inspiratory and expiratory HRCT	16
Lee, K.-N. et al	2000	Korea	54	37 (68.5%)	48 (24–76)	Retrospective	Paired inspiratory and expiratory HRCT	-
Worthy, S. A et al	1998	Canada	23	12 (52.1%)	60 (28–80)	Retrospective	Paired inspiratory and expiratory HRCT	-

n: sample size; HRCT: high radiation computed tomography

*quantification in pack-years

The HRCT image was acquired during full inspiration and expiration in a supine position, with analogous window width and window level parameters in all studies. The anatomical level of image capture in each respiratory phase was reported in two studies and was similar, with end-inspiration images obtained at 10-mm intervals from the lung apex to the base and end-expiration images obtained at the five predetermined anatomical levels [13, 15]. In all cases, lesion measurement was performed at corresponding levels in both inspiratory and expiratory CT scans. Adequate expiratory effort was defined as the decrease in the anteroposterior or transverse chest diameter in expiratory CT compared to inspiratory CT, in percentage points, and was an inclusion criterion in all three. studies. In two investigations [13, 14], patients who made an expiratory effort of at least 4% were included, and in one study [15], the threshold was 5%. Two articles documented the use of histopathological examination to confirm CLD in each patient as well as the participation of two independent radiologists in the analyses [13, 14] Further details about the methodology are described in Table 2.

**Table 2:** CT characteristics in the assessment of cystic lung disease.

Study author	Position	Maneuver	Level (I)	level (E)	Window width (HU)	Window level (HU)	Adequate effort (%)	N°. Of radiologists	Histopathological analysis
Hochhegger, B et al	supine position	full I/E	NR	NR	1000–1500	-700 to -600	4	2	Yes
Lee, K.-N. et al	supine position	full I/E	10 mm intervals*	5 selected levels	1000–1500	-700 to -600	4	2	Yes
Worthy, S. A et al	supine position	full I/E	10 mm intervals*	5 selected levels	1000–1500	-700	5	NR	NR

I/E: inspiratory and expiratory; I: inspiration; E: expiration; HU: Hounsfield units, NR: Not Reported

*10 mm intervals from lung apices to the bases

Several true CLDs and lesions that mimic them were addressed in the articles, with honeycombing being the only common diagnosis evaluated by all of them. Hochhegger et al. [14] assessed changes in cystic size in PLCH, honeycombing, and paraseptal emphysema. Lee et al. [13] had three common studies with the previous one but also included centrilobular emphysema, LAM, and cystic bronchiectasis. Worthy et al. [15] considered five diseases (Honeycombing, LAM, bronchiectasis, cystic adenomatoid malformation, and bullae) and were the only one that did not present specific comparison measurements for inspiratory and expiratory CT scans or the average reduction in chest diameter.

Three types of cysts were analyzed in both Hochhegger and Lee’s studies. In paraseptal emphysema, Lee showed that the size of cysts decreased on average by 16.8%, while Hochhegger found a decrease of 5.2% in expiratory CT. Regarding PLCH and honeycombing, Hochhegger found a reduction of 60.9% and 47.5%, respectively, in lesion diameter, while Lee found a reduction of 32.2% and 35.4%. However, Lee’s study has a reduced sample number for PLCH and honeycombing, with three and nine patients analyzed, respectively, while Hochhegger has a larger sample, evaluating 23 and 27 patients. The other true CLD included by Lee, LAM, showed an average diameter reduction of 42.8%, decreasing from 4.9 mm during inspiration to 2.8 mm during expiration. Centrilobular emphysema followed a similar trend to paraseptal emphysema, with a lesser degree of variation between respiratory phases, demonstrating an average reduction of 24.6%. Cystic bronchiectasis decreased from an inspiratory mean of 13.4 mm to 8.7 mm during expiration, resulting in a 35% reduction.

Worthy et al. assessed a total of 23 patients, reporting that most cysts decreased in size during expiration (21 out of 23 patients). The study evaluated patients with honeycombing, bullae, lymphangioleiomyomatosis, bronchiectasis, and cystic adenomatoid malformation, but it does not provide the average reduction in cyst diameter for each etiology. The number of cysts in each case varied from a single cyst to 60% of the lung being occupied. In one of the 7 patients with bullae, the cysts remained unchanged in size during expiration, and in one patient, a single cyst increased in size due to cystic adenomatoid malformation. The mean change in the size of the largest cyst was 19% for observer 1 and 23% for observer 2, while the mean change in size for the smallest cyst was 31% and 26%, respectively [15]. Table 3 provides a more comprehensive overview of the diagnosis covered in each article as well as the findings.

**Table 3:** Comparing inspiratory and expiratory CT scans for the diagnosis of cystic lung disease.

Study author	Year	Region	n	Mean diameter decrease (%)	Lesion number (total)	Diagnose	Size of Cysts			Inner thoracic diameter: mean (SD)
							Inspiration: mean (SD)	Expiration: mean (SD)	δ (%)	
Hochhegger, B	2022	USA	72	19,4	216	PLCH* (n = 23)	13.3 (4.7)	5.2 (2.5)	60.9	9.8 (3.9)
et al						Honeycombing (n = 22)	8.2 (1.2)	4.3 (1.3)	47.5	7.5 (2.8)
						Paraseptal emphysema (n = 27)	19.0 (2.3)	18.0 (2.1)	5.2	6.2 (2.6)
Lee, K.-N. et al	2000	Korea	54	30,6	270	PLCH* (n = 3)	5.9 (2.0)	4.0 (2.0)	32.2	15.3 (4.2)
						Honeycombing (n = 9)	11.0 (4.5)	7.1 (4.5)	35.4	7.2 (1.4)
						Paraseptal emphysema (n = 16)	23.1 (11.5)	19.2 (10.1)	16.8	4.3 (0.2)
						Centrilobular emphysema (n = 9)	15.8 (4.2)	11.9 (4.2)	24.6	5.3 (1.1)
						LAM* (n = 4)	4.9 (1.8)	2.8 (1.3)	42.8	8.7 (2.5)
						Cystic Bronchiectasis (n = 13)	13.4 (4.0)	8.7 (2.9)	35.0	5.7 (1.2)
Worthy, S. A et al	1998	Canada	23	largest cysts:	27	Honeycombing (n = 11)	No specific values were presented for each cyst or patient.			
				4.6 (observer 1)						
				6.2 (observer 2) smallest cysts: 1.4 (observer 1) 0.8 (observer 2)		LAM* (n = 2)				
						Bronchiectasis (n = 6)				
						CPAM* (n = 1)				
						bullae (n = 7)				

n: sample size; PLCH: Pulmonary Langerhans Cell Histiocytosis; LAM: Lymphangiomyomatosis; CPAM: Congenital pulmonary airway malformation; SD: Standard Deviation; δ: Percentage of respiratory changes

* True cystic lung diseases

Using the updated QUADAS-2 tool (S2 File), we further evaluated the studies’ quality and bias risk. In the "patient selection" category, all studies were considered to have a low risk of bias. In the “index test” and “reference standard” domains, two studies were at low risk of bias, and one was unclear. Furthermore, regarding “flow and timing”, two studies were categorized as having a low risk of bias, and one was classified as high risk.

## Discussion

In this systematic review, we analyzed three studies that performed paired inspiratory and expiratory CT scans to assess and compare the size of lesions during different respiratory phases in patients with CLDs, aiming to identify an additional tool to refine their differential diagnosis.

When considering the three types of lesions analyzed in both Hochhegger and Lee’s studies, it is noted that PLCH and honeycombing became considerably smaller during expiratory CT scans, while the size of paraseptal emphysema tended to remain constant during respiratory cycles. Additionally, Lee’s study observed that centrilobular emphysema exhibited a similar pattern to paraseptal emphysema, remaining relatively stable throughout the respiratory cycle, while LAM and cystic bronchiectasis demonstrated significant reduction.

This reduction in size may be attributed to airway-cyst communication, with higher degrees of variation reflecting a greater connection. These findings suggest that expiratory CT can serve as a valuable adjunctive tool in the radiological differential diagnosis between emphysema and other types of diseases with cystic patterns. However, the ability of this method to further narrow the differential diagnosis still requires extensive investigation, with evidence supporting the use of expiratory scans in this field remaining preliminary.

The use of expiratory CT in the assessment of CLDs was first reported in 1991 by L. Marti-Bonmati et al. [16]. In this investigation, the authors performed image acquisition during full expiration to differentiate bullae and cystic bronchiectasis in three patients based on the degree of air trapping, given the challenge of distinguishing these lesions in the inspiratory phase. Subsequently, two additional case reports demonstrated the application of the technique in a patient with honeycombing [17] and in pulmonary cysts related to eosinophilic granuloma and tuberous sclerosis [18], reinforcing the notion that the association between pulmonary cysts and air trapping can be assessed and varies according to the pathology under investigation. More recently, in 2020, Suzuki et al. [19] developed a cyst-airway communication index considering the low-attenuation area relative to the total lung volume at inspiration and expiration to estimate the extent of communication between cysts and airways. The method proved useful in distinguishing BHDS from other diseases.

A pertinent concern when incorporating an additional acquisition into the tomographic study involves the exposure to radiation dose, especially in high-resolution computed tomography (HRCT), which is the technique of choice for diagnosing lung diseases and conducting paired analysis. In light of this matter, it is noteworthy that protocols for paired inspiratory and expiratory CT scans without increasing radiation exposure have already been created. A protocol with this approach was publicated by Hiroto Hatabu and Mizuki Nishino in 2003 [20]. The methodology employs an 8- or 4-detector CT scanner to capture contiguous images with a 1.25 mm thickness during both inspiration and expiration, proving useful in identifying conducting airways associated with air trapping regions and providing insightful information about the extent and distribution of trapped air in the lungs.

This systematic review has several limitations, primarily due to the small number of studies, with only two providing the mean reduction in cyst size for each disease, making it difficult to compare and generalize the findings. Additionally, the selected studies exhibited considerable heterogeneity in sample characterization and diseases assessed and had small sample sizes, probably due to the rarity of the diseases.

In conclusion, this study has suggested that paired inspiratory and expiratory CT scans can be valuable for helping differentiate between emphysema and other diseases with a cystic pattern due to their ability to reveal dynamic properties of the lesions. However, the average reduction in cyst size as a single parameter, as utilized in the studies included in this review, is not sufficient for further refining diagnostics. Therefore, studies employing more advanced techniques, such as those presented by Suzuki et al., emerge as potential opportunities to explore the cyst-airway communication hypothesis and further improve the diagnostic accuracy of paired methods.

## Supporting information

S1 Checklist PRISMA 2020 checklist. (DOCX)

S1 File Full search strategies for all databases. (PDF)

S2 File QUADAS-2 (Quality Assessment of Diagnostic Accuracy Studies) for quality assessment. (PDF)

S1 Data (XLSX)
