# Supplementary material for: Unlocking the clinical potential of paired inspiratory and expiratory CT scans in the differential diagnosis of cystic lung diseases: A systematic review
Source: PLoS One. 2024 Dec 3;19(12):e0314572. doi: 10.1371/journal.pone.0314572 (PMC11614234; doi:10.1371/journal.pone.0314572)
Supplement: S1 File — (PDF) [file pone.0314572.s002.pdf]

S1 File. Full search strategies for all databases.

PUBMED: 23

((((((expiratory CT[Title/Abstract]) OR (expiratory computed tomography[Title/Abstract])) OR (Paired inspiratory-expiratory[Title/Abstract])) OR (expiratory lung[Title/Abstract])) OR (expiratory multisection CT[Title/Abstract])) OR (expiratory chest CT[Title/Abstract])) OR (expiratory[Title/Abstract])) AND (((Cystic lung disease[Title/Abstract]) OR (Cystic Lesions[Title/Abstract])) OR (Cystic lung lesions[Title/Abstract]))

Scopus: 0

TITLE-ABS-KEY("expiratory CT" OR "expiratory computed tomography" OR "Paired inspiratory-expiratory" OR "expiratory lung" OR "expiratory multisection CT" OR "expiratory chest CT” OR “expiratory") AND TITLE-ABS-KEY("cystic AND lung AND disease OR cystic AND lesions OR cystic AND lung AND lesions")

BVS: 42

((expiratory CT) OR (expiratory computed tomography) OR (Paired inspiratory-expiratory) OR (expiratory lung) OR (expiratory multisection CT) OR (expiratory chest CT) OR (expiratory)) AND ((Cystic lung disease OR Cystic Lesions) OR (Cystic lung lesions))

Embase: 5

('expiratory ct':ti,ab,kw OR 'expiratory computed tomography':ti,ab,kw OR 'paired inspiratory-expiratory':ti,ab,kw OR 'expiratory lung':ti,ab,kw OR 'expiratory multisection ct':ti,ab,kw OR 'expiratory chest ct':ti,ab,kw) AND ('cystic lung disease':ti,ab,kw OR 'cystic lesions':ti,ab,kw OR 'cystic lung lesions':ti,ab,kw)

Cochrane: 44

(expiratory ct OR expiratory computed tomography OR paired inspiratory-expiratory OR expiratory lung OR expiratory multisection ct OR expiratory chest ct OR expiratory) AND (cystic lung disease OR cystic lesions OR cystic lung lesions) in Title Abstract Keyword - (Word variations have been searched)
