# Supplementary figures and images for: Unlocking the clinical potential of paired inspiratory and expiratory CT scans in the differential diagnosis of cystic lung diseases: A systematic review
Source: PLoS One. 2024 Dec 3;19(12):e0314572. doi: 10.1371/journal.pone.0314572 (PMC11614234; doi:10.1371/journal.pone.0314572)

**S2 File. QUADAS-2 (Quality Assessment of Diagnostic Accuracy Studies) for quality assessment.**

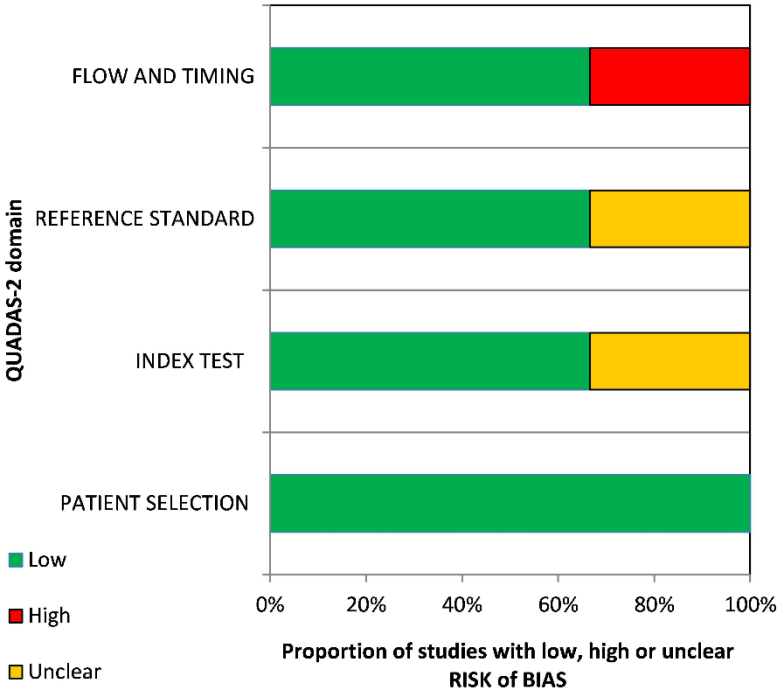

Supplement: S2 File — (PDF) [file pone.0314572.s003.pdf]
